# How to Harness Education Fellows to Optimise Clinical Placement Capacity

**DOI:** 10.1111/tct.70061

**Published:** 2025-03-09

**Authors:** Mattie Williams, Shuchi Kohli, Pamela Leventis

**Affiliations:** ^1^ Maidstone and Tunbridge Wells NHS Trust London UK; ^2^ Kent and Medway Medical School Canterbury UK

## Abstract

There is a global need to increase our medical workforce to meet the demands of changing population demography and increased complex comorbidity. Recruitment to medicine, nursing and other healthcare professions must significantly increase [1]; however, clinical placement saturation is becoming a rate‐limiting step to substantial increase in student numbers. This article will consider how clinical placement providers can deploy education fellows (EFs) to innovatively create capacity whilst enhancing undergraduate clinical placement quality. Clinical teaching fellows are increasingly becoming pivotal players in supporting delivery of undergraduate clinical education and promoting student experience. Unlike clinical teaching fellows, EFs do not have clinical commitments and can reliably coordinate, create and deliver diverse undergraduate learning, mentoring and assessment activity, alleviating senior clinical staff of competing educational responsibilities. We propose that integrated EF‐led innovation can facilitate more consistent, high‐quality placement learning whilst growing placement capacity, without compromising patient care. This article focuses on educational interventions within hospital clinical placements for students from a range of healthcare disciplines including medicine, nursing and allied healthcare professions. The change in workforce requirements and consequent need for a greater undergraduate capacity should catalyse wider discussion around the structure of healthcare education and philosophies of placement development. The authors' experience is in medical education; however, the principles are widely applicable to all healthcare professions for whom experiential placement learning is a necessary training component. Reimagining and restructuring of placement experiences will support healthcare faculty, universities and regulators in supporting sustainable health workforce expansion and equitable healthcare access for all.

## 1 Introduction

The global healthcare workforce needs to grow rapidly and sustainably to meet the demographic and epidemiological demands of all healthcare economies. The 2030 goals outlined by the World Health Organisation (WHO) emphasise the need for equitable access to skilled healthcare workers and enhanced capacity to train more healthcare professionals and sustain workforce density despite population growth, ageing and workforce migration [1]. In 2020, the WHO estimated a shortage of 15 billion healthcare workers, particularly in rural, remote areas [2]. Many countries have initiated plans to significantly expand and reform medical education to enhance recruitment and training of healthcare professionals of all disciplines to optimally address the needs of patients with chronic diseases and strengthen community care [3, 4]. However, whilst local healthcare training programmes will eventually benefit underserved communities, stretched clinical resources pose challenges to immediate placement capacity, supervision and teaching [5].

Insufficient clinical placement capacity is a key risk to proposed expansions in healthcare student numbers. Indeed the experience borne out by many universities is that local placement capacity is already saturated, with little reserve for additional students without compromising patient safety or learner experience [6]. To accommodate the necessary expansions in healthcare workforce training, the shape and content of traditional clinical placements must be innovatively reimagined to provide greater capacity without compromise to patient care. This article describes how the recruitment of education fellows (EFs) can robustly support design, delivery and quality assurance of clinical placements whilst providing diverse learner experiences which simultaneously increase placement capacity.

The shape and content of traditional clinical placements must be innovatively reimagined.

## 2 The Role of Education Fellows

Teaching fellow roles can be undertaken by healthcare professionals at any stage of their career and usually include clinical work, often termed clinical teaching fellows; however, we refer to fully educational roles with no clinical responsibilities filled by graduates within 5 years of primary qualification. In addition to teaching, EFs may act as mentors, examiners and authors of teaching content and examination questions. The authors have experience of recruiting an annual team of eight full‐time EFs to support the local undergraduate medical education programme in a hospital trust that hosts more than 150 medical students at any one time.

**FIGURE 1:**
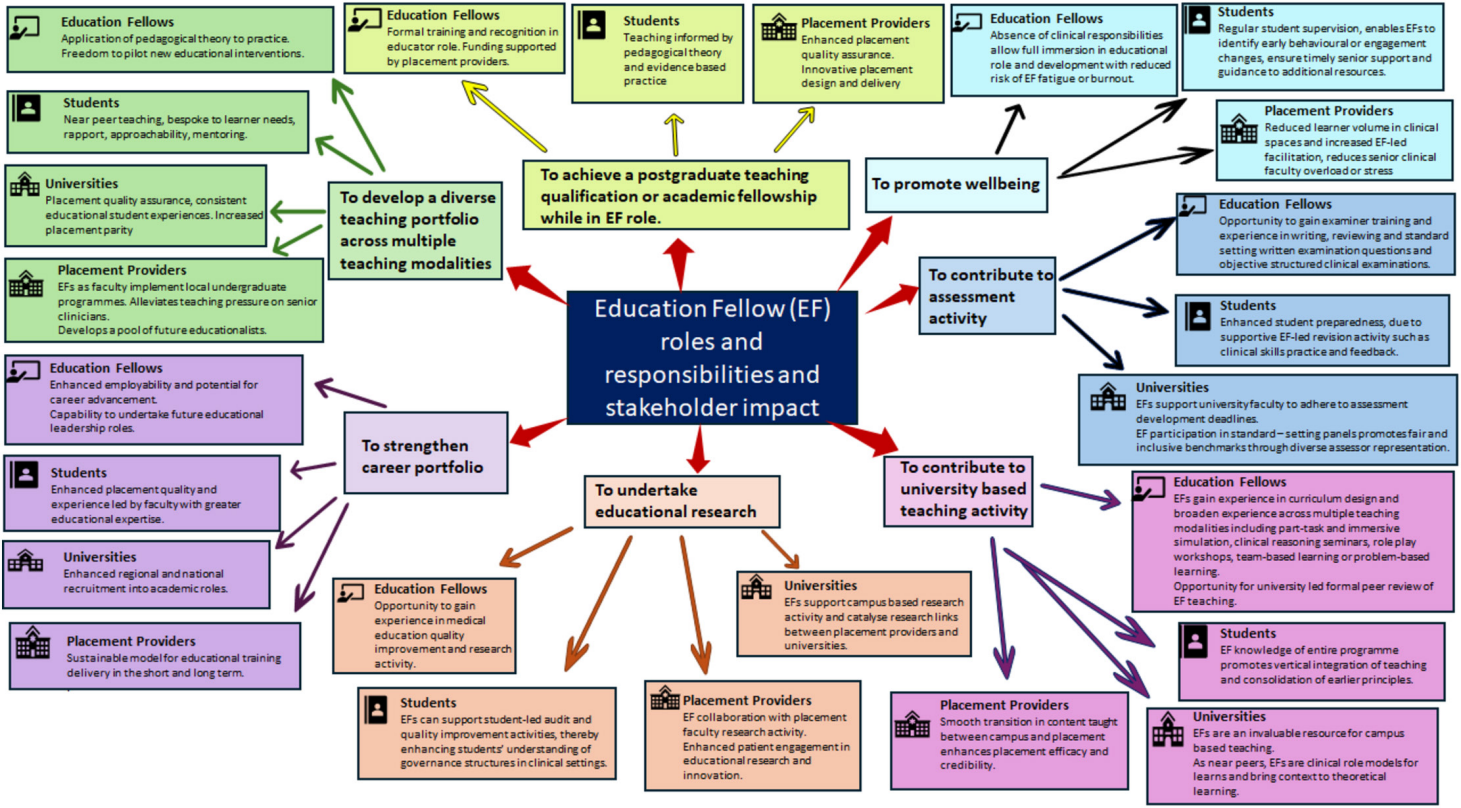
Mind map summarising the roles and responsibilities of education fellows and the impact on stakeholders.

EF posts are mutually beneficial to post holders and placement provider organisations, universities, students and patients, with evidence to show that they can lead high‐quality clinical placement delivery [7]. Recently graduated healthcare professionals are closer to students' stage of training, which enhances their rapport, relatability and approachability, thus developing student confidence and professional identity [7]. Both medical and nursing students have cited the value of educational fellows in scaffolding the transition from university to placement and placement to practice, both of which can be daunting prospects for many [8, 9]. The absence of clinical activity optimises reliability and engagement, whilst allowing EFs to diversify learning opportunities, spend more time with individual learners and provide tailored teaching and feedback. Importantly, EFs bring stability, consistency and reliability to the busy, sometimes unpredictable clinical learning environment. Figure 1 summarises the broad roles and responsibilities of EFs and the beneficial impact on their own development as well as that of students, universities and healthcare providers. Highlighting the extensive, versatile value of these posts can fortify local business cases and trust, community and university‐led initiatives to make funding available for these positions.

EFs bring stability, consistency and reliability to the busy, sometimes unpredictable clinical learning environment.

## 3 Clinical Placements

Clinical placements vary in structure globally and between healthcare professions. However, the broad principles of students attending clinical spaces to build an experiential portfolio of clinical skills, caseloads and interactions to develop their clinical acumen are largely universal. As healthcare service pressures increase in parallel with undergraduate numbers, measures must be taken to increase teaching capacity and implement innovations to at least maintain but ideally improve placement quality. EFs are potentially pivotal players in facilitating placement reform.

Within existing placement structures, learners attend various clinical activities and engage in observation, shadowing and supervised practice of clinical skills through information gathering, physical examinations and procedures. Placement learning is inherently serendipitous. Taking an example from medical education, one learner may observe multiple patients with chronic obstructive pulmonary disease, whilst another may see a patient with idiopathic eosinophilic pneumonia. Both are expected to have similar knowledge of all core conditions, presentations, diagnoses, management and complications. In short, luck can inform experiential learning for healthcare students on clinical placement. By increasing the number and scope of EFs, it is possible to reduce the degree to which chance dictates learners' experiences and provide greater consistency across student cohorts.

It is possible to reduce the degree to which chance dictates learners' experiences and provide greater consistency.

A further problem encountered by students on placement is over‐saturation of clinical areas with risk of overwhelming healthcare staff and patients. EFs can help address this issue directly through diversifying time spent on clinical placement without dilution of focus on necessary learning outcomes. Examples of innovative EF‐led educational initiatives to support this are shown in Tables 1 and 2 and Boxes 1 and 2. This approach can offset the barriers to increased student numbers on placement and ensure standardised, safe and engaging teaching interventions alongside timetabled sessions in clinical areas. This facilitates maximum capacity across combined clinical and teaching spaces. The activities listed in Tables 1 and 2 reflect the authors' experiences in the context of medical acute trust placements, but can be expanded, modified and developed across unlimited portions of undergraduate healthcare placement programmes to supplement direct patient‐facing learning. Multiple innovative opportunities for interprofessional education are described. Table 1 lists seminars and workshops that could be initiated without extensive financial outlay or infrastructural change, whilst Table 2 illustrates simulated and virtual interventions which may require technological investment, expertise and planning, two of which are further described as case studies in Boxes 1 and 2.

**TABLE 1:** Examples of clinical placement EF‐led seminars and workshops.

Educational intervention	Background
	Role of EF
Standardised topic‐based seminars	Regular seminars with consistent materials delivered by EFs and supplemented by EF‐led bedside teaching. Standardised content ensures parity in student teaching.
	EFs create topic‐based teaching materials to supplement clinical exposure across range of specialties. They facilitate small group teaching and identify suitable inpatients to consolidate learning.
Prescribing workshops	Students receive simulated patient notes and apply clinical reasoning to diagnose and prescribe according to diagnoses, comorbidities, medications and allergies. The workshops can include polypharmacy, medication reviews and deprescribing. This activity can be inter‐professional, alongside pharmacy students.
	EFs create resources and facilitate workshops. They review completed drug charts and provide feedback to support safe and accurate prescribing.
Expert patient seminars	Patients living with long‐term conditions are invited to inter‐professional student seminars to share their lived experience. Formats may include presentations such as ‘a week in the life of’ or questions posed by students allowing wider discussion.
	EFs organise and facilitate seminars and support student reflection on common threads between chronic diseases and the wider impacts on individuals and carers.
Interprofessional education workshops	Medical, nursing and allied health professional students discuss patient cases and work through structured assessment and decision making. This fosters mutual understanding of skills, perspectives, roles and responsibilities of multidisciplinary team members, and how to collaborate efficiently to provide patient centred care.
	EFs author cases and question design and co‐facilitate alongside other multi‐disciplinary faculty.
Inclusivity/decolonisation seminars	Medical, nursing and allied healthcare professional students attend an equality, diversity and inclusion (EDI) seminar series to promote case‐based discussion and reflection on topics such as microaggressions, unconscious bias, attainment gaps and health inequalities [10].
	EFs develop seminar materials and facilitate each themed seminar.
Patient safety, workshops	Inter‐disciplinary workshops allow students to apply policy to practice in the context of patient safety scenarios. Cases may include reporting, the impact of errors the duty of candour, learning from errors and their diverse etiologies.
	EFs create structured patient safety scenarios and collaborate with multidisciplinary healthcare faculty to facilitate the workshops.

**TABLE 2:** Examples of clinical placement EF‐led virtual or simulated activities.

Educational intervention	Background
	Role of EF
Clinical skills case‐based teaching workshop	Students follow a fictional patient journey from initial presentation and perform simulated clinical skills to facilitate full assessment, diagnosis and initial management. The workshop can integrate revision of multiple procedural skills such as venepuncture, cannulation, catheterisation, nasogastric tube insertion, arterial blood gas sampling as well as prescribing and contextual data interpretation.
	EFs create the patient journey and facilitate and debrief the workshop with individual learner feedback.
Virtual emergency department triage and review	Students discuss, triage and prioritise a series of cases as if in an emergency department, before deciding upon investigation and management options [11].
	EFs develop materials, facilitate sessions and feedback on simulated referrals.
Virtual on‐call (see Box 1)	Penultimate and final year students are placed in a simulated on‐call environment. Further detail is provided in Box 1 case study.
	EFs create scenarios and patient records and facilitate virtual on‐call and debrief.
Simulation scenarios	Structured simulated clinical scenarios ensure consistent exposure to common or serious presentations in a safe environment. The benefits of high fidelity simulation are well described [13]. Simulation can be interprofessional allowing medical, nursing and allied healthcare students to participate appropriate to role and stage.
	EFs support all aspects of simulation teaching including writing scenarios, facilitating and participating in the simulation and facilitating debriefs.
Medical escape room (see Box 2)	Medical escape rooms are described further in Box 2 case study.
	EFs create and deliver medical puzzles within an escape room setting.
Theatre live streamed session	Audiovisual links from operating theatres to teaching spaces allow live streaming of chosen surgical procedures to EF facilitated observing students. The operating field is visible whilst the lead or assisting surgeon narrates the procedure [15].
	EFs support students throughout the live‐streamed surgery. They may facilitate responses to questions from students or pose these to the operating team.
Live‐streamed ward rounds/outpatient clinics	Students observe clinical interactions from a larger EF facilitated space. The ward round or outpatient consultation is live streamed with investigations. ‘HoloLens 2’ software can project blood tests and radiological images adjacent to the patient [16].
	EFs support students throughout and encourage application of clinical reasoning to develop differential diagnoses and management plans.

Box 1 **Virtual on‐call case study.**

The virtual on‐call is a simulated activity taking place on wards within the placement hospital. Students are provided with a pager which facilitators use to contact them and ask to complete paper‐based tasks left for them in clinical locations such as ward nursing stations. The focus is on clinical task prioritisation, note‐taking, handover and appropriate escalation skills. This allows students to experience simulated on‐call work in a safe environment, receive constructive feedback and engage in group discussions and reflection.

We deliver weekly sessions over a four‐week programme allowing students to rotate through four sets of five scenarios which are designed and run by EFs to simulate common on‐call tasks. Students are also sent ‘distractor’ pager messages to allow them to triage appropriate and inappropriate tasks, before prioritising clinical tasks in order of completion. Tasks are organised to be incrementally more complex in order to continually challenge students and stretch the scope of their learning.

In order to maintain the quality and safety of patient care, an emphasis is placed upon a safe clinical learning environment, underpinned by the concept of ‘psychological safety’ for students. Students are always able to escalate to seniors for support and reassurance is given during debrief sessions that information is confidential, no criticisms are made but learning is through constructive peer feedback and open discussion.

Virtual on‐call sessions have been found to increase students' confidence in essential skills required for clinical work and overcome fears to help ease the transition into practice [12].

Box 2 **Medical escape rooms case study.**

Medical escape rooms are highly effective simulation‐based activities which can be planned and mapped to required learning outcomes [14]. The concept is well documented in the literature and can be successfully utilised across healthcare professions [14]. Blending simulation with escape room techniques adds an element of fun and ‘game‐playing’, reducing learner anxiety during the experience, whilst simultaneously increasing educational value through experiential learning [14].

We have created and executed five themed medical escape rooms each accommodating five to seven medical students, allowing for around 30 learners per escape room theme every 6 weeks throughout the academic year. We accommodate full cohorts of students on clinical placement, with some choosing to attend multiple sessions. Each scenario lasts approximately 45 min with the ultimate goal of ‘escaping’ the simulated environment. Learners must complete a focused clinical assessment, with appropriate senior escalation and correct management plan for their stage. We ensure activities are enjoyable by combining core presentations, including clinically important simulated signs with creative puzzles. For example, on entering the room, students may elicit simulated stridor in the patient mannequin and attempt to remove the visible foreign body, which is a written paper clue inviting them to consider next steps. Puzzles vary from arithmetic calculations and letter decoding to props holding clues. Clinical scenarios were adapted from EFs previous clinical experiences and ensure exposure to a variety of medical specialties. Limitations to medical escape rooms include the cost of high fidelity simulated mannequins, however the concept of puzzles and systematic assessment remains applicable with low‐fidelity mannequins.

EFs complete a local ‘training the trainer’ programme and are competent simulation facilitators. They coordinate the entire medical escape room activity including:

- Planning the scenarios and clinical assessments
- Developing creative clues to maintain learner engagement
- Piloting scenarios with resident doctors to ensure feasibility
- Facilitating the escape room scenario, ensuring learners systematically approach problem‐solving, providing additional clues if needed.
- Debriefing the scenario addressing technical and non‐technical aspects, often with a focus on communication, teamwork and other human factors

Students are often already familiar with the EFs and thus more inclined to ask questions, further enhancing psychological safety throughout the activity. Whilst the majority of learners are medical students and doctors in their first year of residency training; this structure can also be applied to interprofessional scenarios.

## 4 Limitations and Wider Considerations

Classroom and technology enhanced innovations allow student cohorts to experience and learn from more standardised sets of patient cases. Whilst this augments knowledge, skills and familiarity, there are potential limitations to these modalities which are largely remote from the immediate clinical space. Students cannot practice one‐to‐one communication and physical examination skills with real patients, under direct supervision. Reduced time in the clinical space also impacts the development of other professional skills and behaviours, many of which cannot be measured or assessed through written or practical examinations. It is therefore crucial that medical educators curate methods to harness the benefits of innovative educational activities adjunctively with direct patient contact, thus providing students with the optimal placement experience.

We must also consider the ethical and patient safety implications of innovations such as live‐streaming [16] and how patient confidentiality and data protection can be secured. Many patients may be uncomfortable for their procedure or consultation to be live streamed across to an unseen cohort of students and informed consent must be sought. Recording such events would raise additional concerns around storage, longevity and the need for measures to prevent downloading, photography or sharing of content designed for placement teaching.

## 5 Future Developments

The clinical placement enhancement strategies and interventions described in this article focus upon EF‐led initiatives, whereby materials and virtual resources are created to supplement time spent with patients and healthcare professionals in clinical environments. However, generative artificial intelligence is becoming increasingly embedded into undergraduate and postgraduate education as well as clinical practice [17]. It is entirely possible for patient cases, clinical scenarios, quizzes, images and videos to be created through generative language models. This would allow further exploration of novel, authentic placement teaching methods to be developed, with potentially greater yield from smaller numbers of faculty.

## 6 Integrating EF‐Led Placement Innovation

Many of the EF‐led interventions outlined here can enhance placement capacity whilst minimising reliance upon chance or opportunistic experience to ensure all students have systematically met core clinical presentations thus promoting parity in knowledge and skills at graduation. A number of innovations lend themselves to interprofessional sessions and can also be converted into workplace‐based assessments and form part of undergraduate portfolios alongside other more traditional clinical patient‐facing activities.

Time spent on clinical placement and regular close engagement with clinicians, nurses and allied healthcare professionals supports students to develop as future healthcare professionals whilst they are also honing their clinical skills and knowledge. It is essential that students continue to benefit from regular immersion and socialisation in the multidisciplinary clinical environment, thus developing professional identity [18]. However, placement experiences can be diversified, augmented and standardised by EF‐led innovation.

Placement experiences can be diversified, augmented and standardised by EF‐led innovation.

## 7 Conclusion

Successful placement providers must be capable and ready to adapt. Widespread expansion of healthcare student intakes will be necessary to meet global growing workforce demands and a fresh review of placement design and delivery is therefore pressing. EFs can be key facilitators in alleviating pressure on clinical environments whilst developing tomorrow's healthcare professionals and adding to the pool of trained clinical faculty. Timetabling innovative and structured placement experiences alongside traditional opportunistic clinical encounters can simultaneously enhance and standardise student exposure.

EFs can be key facilitators in alleviating pressure on clinical environments whilst developing tomorrow's healthcare professionals.

We propose that placement providers invest in fully educational fellow roles, free of clinical responsibility to allow dedicated attention to thoughtful, innovative placement design and delivery. Future successful clinical placements should be redefined to include a combination of attendance and engagement in clinical environments supplemented by a complementary selection of EF‐led innovations.

## Author Contributions

**Mattie Williams:** conceptualization, writing – original draft, writing – review and editing. **Shuchi Kohli:** conceptualization, writing – original draft, writing – review and editing. **Pamela Leventis:** conceptualization, writing – original draft, writing – review and editing, supervision, project administration.

## Conflicts of Interest

The authors declare no conflicts of interest.

## Data Availability

Data sharing is not applicable to this article as no new data were created or analyzed in this study.
